# Supplementary material for: Venomix: a simple bioinformatic pipeline for identifying and characterizing toxin gene candidates from transcriptomic data
Source: PeerJ. 2018 Jul 31;6:e5361. doi: 10.7717/peerj.5361 (PMC6074769; doi:10.7717/peerj.5361)
Supplement: Supplemental Information 3 [file peerj-06-5361-s003.gz › FinalOutput_E-6/Kunitz-type_serine_protease_inhibitor_homolog_beta-bungarotoxin_B5-B_chain_1/finaltree.pdf]

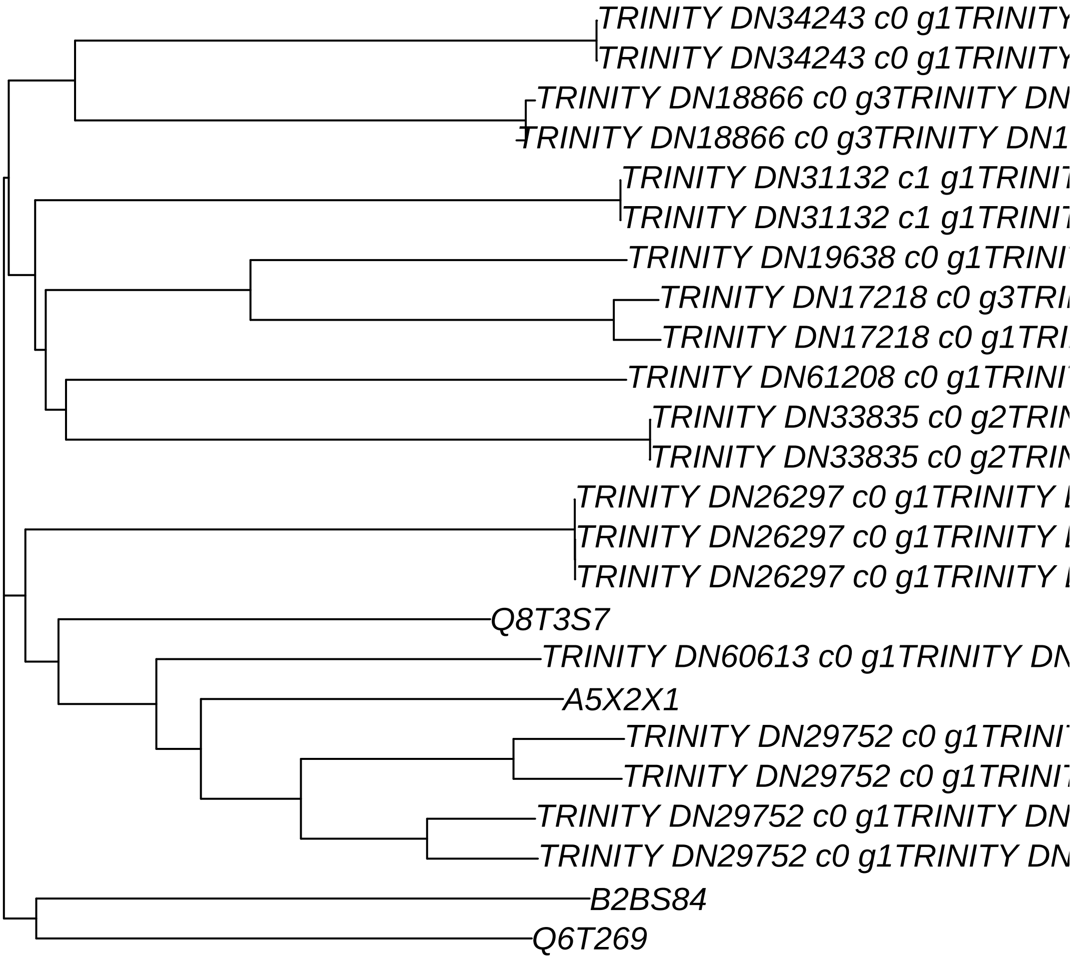
TRINITY DN34243 c0 g1TRINITY
TRINITY DN34243 c0 g1TRINITY
TRINITY DN18866 c0 g3TRINITY DN
TRINITY DN18866 c0 g3TRINITY DN1
TRINITY DN31132 c1 g1TRINIT
TRINITY DN31132 c1 g1TRINIT
TRINITY DN19638 c0 g1TRINI
TRINITY DN17218 c0 g3TRI
TRINITY DN17218 c0 g1TRI
TRINITY DN61208 c0 g1TRINI
TRINITY DN33835 c0 g2TRIN
TRINITY DN33835 c0 g2TRIN
TRINITY DN26297 c0 g1TRINITY
TRINITY DN26297 c0 g1TRINITY
TRINITY DN26297 c0 g1TRINITY
Q8T3S7
TRINITY DN60613 c0 g1TRINITY DN
A5X2X1
TRINITY DN29752 c0 g1TRINIT
TRINITY DN29752 c0 g1TRINIT
TRINITY DN29752 c0 g1TRINITY DN
TRINITY DN29752 c0 g1TRINITY DN
B2BS84
Q6T269
